# Antineutrophil cytoplasmic antibody associated vasculitis: a converging diagnosis from neuropathy and testicular infarcts

**DOI:** 10.1093/jscr/rjac548

**Published:** 2022-11-26

**Authors:** Ross Mercer, Angus White, Christopher Bates

**Affiliations:** School of Anatomy, University of Bristol, Bristol, UK; Department of Urology, Aneurin Bevan University Health Board, Newport, UK; Department of Urology, Aneurin Bevan University Health Board, Newport, UK

## Abstract

Antineutrophil cytoplasmic antibody (ANCA) associated vasculitis encompasses a group of rare multi-system affecting diseases that can present with unremitting cases of common conditions. We present a case of a middle-aged gentleman admitted under Urology with epidymo-orchitits on multiple occasions. Ultrasound revealed segmental testicular infarcts, and he was managed conservatively. He re-presented shortly after discharge to the medical assessment unit with ulnar nerve paraesthesia, thought to be due to ulnar nerve entrapment syndrome. Less than 1 week later he was re-admitted again, with severe peripheral nerve pain in all limbs and multifocal weakness. Initial history and examination gave a provisional diagnosis of mononeuritis multiplex. Following investigations and treatment, this was deemed to be caused by a converging diagnosis of C-ANCA PR3 positive vasculitis, a small vessel vasculitis. This case highlights that patients with unremitting cases of epididymo-orchitis with testicular infarcts may benefit from autoimmune screening.

## INTRODUCTION

Antineutrophil cytoplasmic antibody (ANCA) associated vasculitis encompasses a group of rare small vessel vasculitides, which have multi-system involvement due to inflammation of blood vessels. Presentation can occur at any age and affect 20–25 people per million per year in Europe [1]. Recognized distribution of the multi-system involvement includes a vasculitic rash with systemic features (<20%), respiratory symptoms (≈45%), ear, nose and throat symptoms (≈45%), eye symptoms (<20%), nerve symptoms (≈30%) and renal disease (≈65%; [2, 3]).

Genitourinary symptoms are a less well documented presentation of ANCA vasculitis. In this case, we present an example of a patient presenting with recurrent orchitis and small vessel infarction.

## CASE REPORT

**Figure 1:**
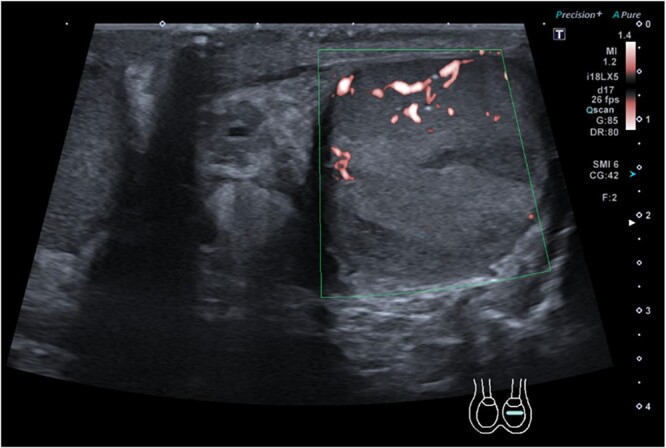
Ultrasound sonography of left testicle demonstrating wedge segmental infarcts which are avascular on colour doppler.

A middle-aged gentleman initially presented to the Urology Assessment Unit with right testicular and abdominal pain. Notable medical history includes irritable bowel syndrome and previous right testicular torsion surgery performed twelve years prior. The patient was started on oral antibiotics for right epididymo-orchitis and discharged home. He re-presented 1 week later with left testicular pain; examination revealed a normal right testicle, scar from previous fixation on the right, left scrotal erythema and testicular enlargement, which was exquisitely tender. Ultrasound sonography (USS) of the testicles showed segmental infarcts of the left testis (Fig. 1), no collection, minor left varicocele and hydrocele; there was normal appearance of the right testis. Antibiotics were commenced, once improved the patient was discharged home with a diagnosis of left necrotising epididymo-orchitis.

Six days later this patient presented to the Medical Assessment Unit with paraesthesia of his left fifth digit, up to proximal interphalangeal joint with gradual increase in sensation mid palm and normal sensation beyond this. There was associated pain in this distribution and weakness of abduction of the fingers. He had been experiencing these symptoms around the day of his last discharge from Urology. Blood results were consistent with the recent epidymo-orchitis and did not prompt an emergent reason for admission to hospital. An incorrect diagnosis of ulnar nerve entrapment syndrome was made, and the patient was discharged.

Less than a week later, he re-presented with significant worsening of his peripheral neuropathy. The paraesthesia in his left fifth digit had spread to include the entire left hand, right hand and distal to the knees. The pain was described as a burning neuropathic pain. He was unable to mobilize and lived alone, so it was decided he required urgent admission. At this point the diagnosis was unclear, but examination pointed towards a preliminary diagnosis of mononeuritis multiplex due peripheral nerve involvement.

Autoimmune screen bloods were performed; this showed a positive ANCA screen (titre 1:160) with C-ANCA pattern. In addition, anti-PR3 antibodies were 1537.7 U/mL (Normal limit <20 U/mL). Bloods also revealed the beginnings of chronic kidney disease. There were no lower or upper respiratory symptoms. Nerve conduction studies provided more support to the diagnosis, suggesting a motor and sensory polyneuropathy. Altogether this unified to a diagnosis of C-ANCA PR3 positive vasculitis.

The patient was commenced on prednisolone and rituximab and the disease began entering remission. Since his discharge from hospital, the patient has been receiving 3 monthly follow up and 4 weekly blood tests to monitor his renal function. He has also received repeat nerve conduction studies, which showed evidence of improvement of both sensation and motor nerves. Despite this, his limb function remained impaired, and he had to use a Zimmer frame and wheelchair to mobilize. His epididymo-orchitis did not require surgery and settled with conservative management, he had no ongoing testicular problems. The mainstay of treatment going forward will be maintaining remission of the disease, physiotherapy and symptom management.

## DISCUSSION

This case demonstrated the association of vasculitis with testicular infarcts, which may be misdiagnosed as simple epididymo-orchitis until systemic involvement presents.

Brimo *et al.* [4] conducted a case series of localized testicular infarction with associated vasculitis including 19 cases. These orchidectomy specimens were subsequently examined for isolated vasculitis and presence of systemic disease, which showed 25% of cases were associated with a systemic vasculitis and upwards of 50% if the testicular specimen were granulomatous. These cases presenting as testicular pain were interpreted as either epididymo-orchitis or cancer, and all of them advanced to testicular infarcts on ultrasound significant enough to prompt orchidectomy. This conclusion advocated for further clinical investigation of systemic vasculitis and could support the use of autoimmune screens following detection of testicular infarcts on ultrasound.

Teh *et al.* [5] demonstrated another case of testicular infarction associated with a vasculitis. This case highlighted that it can be challenging to determine the root cause of a testicular infarction, as it can also be due to infection or hypovascular tumours. As well as vasculitis autoimmune screening, the authors concluded that testicular infarcts should also undergo tumour marker testing.

The National Institute for Health and Care Excellence (NICE; [6]) suggests testicular USS and referral to Urology if there are persistent symptoms over 2 weeks for epididymo-orchitis, however there is no clear guidance on the interpretation and management following the USS report. Throughout this case there was no deviation from the recommended guidelines, but we have clearly highlighted a scope for clinicians to consider initiating autoimmune and tumour marker screening for patients presenting with testicular infarcts on USS.

To conclude, encouraging appropriate early investigations may slow or halt systemic progression of a patient’s vasculitis and prevent lifelong disability. The patient discussed in this case may have benefited from autoimmune screening following testicular infarcts on USS. Current recommendations in the literature support autoimmune screening for unremitting disease processes in certain organ systems but exclude the genitourinary system. More evidence linking testicular infarcts and vasculitis would be required to make autoimmune screening following recurrent epididymo-orchitis or testicular infarct on USS a standardized approach in practice.

## CONFLICT OF INTEREST STATEMENT

None declared.

## FUNDING

None.
